# Characteristics of Vaginal Microbiome in Reproductive-Age Females with HPV Infection in Xinjiang, China

**DOI:** 10.1155/2022/7332628

**Published:** 2022-11-01

**Authors:** Yan Xia, Ying Feng, Tianhua Qin, Xiaohua Zhao, Jing Lu, Cailing Ma

**Affiliations:** ^1^Department of Gynecology, The First Affiliated Hospital of Xinjiang Medical University, State Key Laboratory of Pathogenesis, Prevention and Treatment of High Incidence Diseases in Central Asia, Urumqi 830000, China; ^2^Department of Gynecology, Urumqi Maternal and Child Health Hospital, Urumqi 830000, China; ^3^College of Public Health, Xinjiang Medical University, Urumqi 830000, China

## Abstract

**Objective:**

We investigated the characteristics of vaginal microbiome in reproductive-age females with HPV infection in Xinjiang, China.

**Methods:**

A total of 135 females of reproductive age were enrolled. There were 43 healthy HPV-negative females in control group (*N* group), 58 HPV-positive females in nonlesion group (*P*1 group), and 34 HPV-positive females in low-grade squamous intraepithelial lesion group (*P*2 group). DNA was extracted from the vaginal secretions, and V3–V4 regions of bacterial 16S rDNA were amplified and sequenced by NovaSeq. QIIME2 and R software were used to perform diversity analysis of bacteria. PICRUSt2 was used to predict the function of the vaginal microbiota.

**Results:**

*Lactobacillus* was the main genus of vaginal microbiota in asymptomatic reproductive-age females with or without HPV in Xinjiang. The diversity of vaginal microbiota in the *P*1 group was significantly higher than that in the *N* group, and the proportion of *Gardnerella* increased significantly. The vaginal microbiota structure of the *P*2 group was different from the N group, characterized by the decrease of *Lactobacillus crispatus* and the increase of *Shuttleworthia*. The function of the inordinate microbiome may play a role in accelerating HPV replication and integration.

**Conclusion:**

The structure of vaginal microbiota alters under persistent HPV infection in asymptomatic females of reproductive age in Xinjiang. The *Gardnerella* increase is associated with increased susceptibility to HPV infection, and *Lactobacillus iners* predominance and *Shuttleworthia* presence may be a signature of HPV infection with low-grade squamous intraepithelial lesion.

## 1. Introduction

Cervical cancer (CC) is a common malignant tumor in females, and its incidence is second only to breast cancer in genitourinary system. According to statistics, about 604,000 new cases and 342,000 deaths occurred worldwide in 2020 [1]. Females with sexual experience have an 80% chance of being infected with human papilloma virus (HPV) in their lifetime. Among the infected cases, 70%–80% of them would regress spontaneously in 12 months, and only 10% of them would develop into HPV persistent infection after 24 months, consequently resulting in CIN (cervical intraepithelial neoplasm) and even CC [2]. The vaginal microbiome has become one of the important influencing factors of HPV infection and development of cervical precancerous lesions and CC [3–5].

Clinical studies have observed that the diversity of the vaginal microbiome increases significantly with the severity of cervical precancerous lesions [6, 7]. Mechanistically, it is confirmed [8] that HPV E7 protein reduces the secretion of vaginal mucosal defense peptides, which are the energy source of amino acid metabolism of *Lactobacillus* spp. The decreasing *Lactobacillus* spp. may contribute to vaginal pH increase, which may favor the growth of vaginal pathogenic bacteria and result in structural changes in the vaginal microbiome ultimately. This suggests that HPV can regulate the reproduction of the vaginal microbiome and alter the vaginal environment by inhibiting *Lactobacillus * spp.

After DNA integration into the host genes, the HPV E6/E7 protein is highly expressed, which binds to tumor suppressor gene p53 and Rb to inhibit apoptosis and finally induces CIN and CC [9, 10]. E6/E7 protein/RNA is rarely expressed in the low-grade squamous intraepithelial lesion (LSIL) [11] but is highly expressed in tissues of high-grade squamous intraepithelial lesions (HSIL) and CC [12, 13]. Investigators have paid more attention to the association of vaginal microbiome and grades of squamous intraepithelial lesion and revealed many differences in HSIL and CC. However, the characteristics of the vaginal microbiome in HPV-infected patients with or without LSIL are less known.

Herein, we investigated that if the characteristics of the vaginal microbiome in healthy reproductive-age females and reproductive-age females with HPV infection accompanied with LSIL or not, in Xinjiang, China. Our findings may help the clinical diagnosis and treatment of HPV infection and cervical precancerous lesions.

## 2. Materials and Methods

### 2.1. Study Participants

Female patients who received colposcopy examination in the Urumqi Maternal and Child Health Hospital from April 2021 to October 2021 were selected. Inclusion criteria are as follows: (1) Patients were permanent residents in Xinjiang, China. (2) Patients had a history of sexual life. (3) The age of patients was between 18 and 49 years. (4) Patients were in nonpregnancy and nonmenstrual periods. (5) Patients had regular menstrual cycle, with a cycle of 25–35 days. Exclusion criteria are as follows: (1) Patients had sexual intercourse, vaginal lavage, or vaginal medication within 3 days. (2) Patients took sex hormone within 3 months. (3) Patients took any antibiotics within 1 month. (4) Patients received hysterectomy or traumatic treatment of cervix uterus. (5) Patients were with acute inflammation of the genitourinary system. (6) Patients received immunosuppressive therapy. (7) Patients had complications of severe heart, liver, and kidney insufficiency. (8) Patients had mental disorders. (9) Patients suffered with malignant tumors. Age-matched healthy females who received physical examination during the same period were recruited as control. All participants received HPV testing and thin-layerliquid-based cytology testing (TCT). Participants who were HPV16/18-positive or were positive for the other 12 high-risk HPVs accompanied with the atypical squamous cell of undetermined significance (ASCUS) or above received colposcopy examination and biopsy when it was necessary. This study was approved by the Ethics Committee of Urumqi Maternal and Child Health Hospital (approval number: XJFYLL2021006). All participants signed the informed consent form.

### 2.2. 16S rDNA Sequencing

The secretions were collected with disposable sterile cotton swabs from the posterior vaginal fornix after sufficiently exposing the vagina and cervix by sterile speculum. The swabs were stored at −80°C immediately until bacterial DNA extraction. Total genomic DNA was extracted from the swabs according to the manual of MicroElute Genomic DNA Kit (D3096-01, Omega Bio-tek Inc, Norcross, Georgia, USA). DNA concentration was measured by NanoDrop 2000. The 341F (5′-CCTACGGGNGGCWGCAG-3′) and 805R (5′-GACTACHVGGGTATCTAATCC-3′) were used as primers to amplify V3–V4 hypervariable regions of the bacterial 16S rDNA. The PCR amplification system (25 *μ*L) included 12.5 *μ*L PusionHot Start Flex 2X Master Mix (M0536L, Yitao Biological Instrument Co., Shanghai, China), 2.5 *μ*L upstream and 2.5 *μ*L downstream primer, and 50 ng DNA template. The PCR amplification procedure was as follows: predenaturation at 98°C for 30 s; 35 cycles of denaturation at 98°C for 10 s, annealing at 54°C for 30 s, and extension at 72°C for 45 s; and final extension at 72°C for last 10 min. Then, PCR products were confirmed with 2% agarose gel electrophoresis and purified by AMPure XT beads (Beckman Coulter Genomics, Danvers, MA, USA). The concentration was measured by Qubit (Invitrogen, USA). The size and quantity of amplicon libraries were assessed on Agilent 2100 Bioanalyzer (Agilent, USA) and with the Library Quantification Kit for Illumina (Kapa Biosciences, Woburn, MA, USA), respectively. The DNA library with concentration above 2 nM was considered qualified. Finally, 25 ng qualified library was sequenced by LC-Bio Technology Co., Ltd. (Hangzhou, China) on the NovaSeq 6000 platform.

### 2.3. Sequencing Data Processing and Analysis

We used QIIME2 [14, 15] to extract high-quality sequences. The raw data obtained by sequencing were subjected to overlap splice, quality control, and chimera filtering, and then high-quality clean data were obtained. After dereplication by DADA2 (Divisive Amplicon Denoising Algorithm 2) [16], we obtained amplicon sequence variant (ASV) feature tables and feature sequences. Taxonomic identification of ASVs was performed using Silva (Release 138) database. The *α*-diversity (Chao1 and Shannon index) and *β*-diversity were calculated by R software (V3.4.4), which was also used for graph plotting. Differences in species abundance between groups were detected using Kruskal–Wallis and Wilcoxon rank sum tests. The linear discriminant analysis (LDA) coupled with effect size measurements (LEfSe) online analysis tool (https://www.omstudio.cn/tool/) was used to analyze the bacterial genera differences between groups.

### 2.4. Statistical Analysis

SPSS 20.0 software was used. The measurement data were tested for normality by the Kolmogorov–Smirnov test. Data with normal distribution were expressed as mean ± standard deviation and analyzed with one-way ANOVA. Non-normally distributed data were expressed as median and interquartile range and were compared using multisample nonparametric tests. Enumeration data were expressed as percentages or rates (%) and were analyzed using the chi-square test or Fisher's exact test. *P* < 0.05 was statistically significant.

## 3. Results

### 3.1. Characteristics of Participants

In total, 92 high-riskHPV-positive patients and 43 high-riskHPV-negative healthy females were enrolled in this study. Among the 92 patients, 58 cases were with normal TCT or normal colposcopy or with chronic cervicitis by histopathological diagnosis of biopsy, and they were classified as the *P*1 group. Thirty-four patients with LSIL were classified as the *P*2 group. The 43 HPV-negative healthy females with normal TCT served as the control group (*N* group). There were no significant differences among groups in terms of age, ethnicity, body mass index, number of pregnancies, number of deliveries, phase of menstrual cycle, contraceptive method, age at first sex, number of lifetime sexual partners, frequency of sex in the past year, and smoking status (*P* > 0.05, Table 1). However, significant difference was presented in the HPV infection status between P1 and P2 groups (*P* < 0.05). The P1 group had more patients infected with HPV16/18 types, while the P2 group had more patients with HPV multi-infection.

### 3.2. Sequencing Results

**Figure 1:**
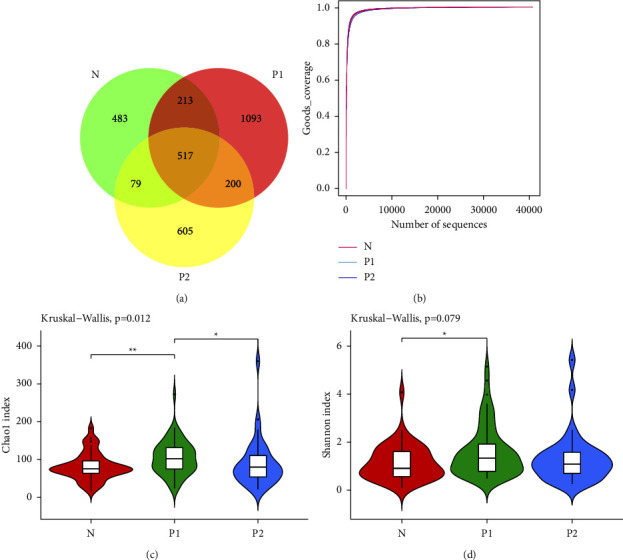
Abundance and diversity of vaginal microbiota. (a) Venn diagram of ASVs in three groups. (b) Sequencing dilution curves of three groups of samples. (c) Violin plot of Chao1 index among three groups. (d) Violin plot of the Shannon index among the three groups. The dark horizontal bar represents the median value of each group, while the boxes represent the 25^th^ and 75^th^ percentile values. ^∗^*P* < 0.05, ^∗∗^*P* < 0.01.

We obtained 10,791,360 assembled clean reads from 135 samples, with an average of 67,572.48 ± 7121.18 reads per sample. A total of 3195 ASVs were obtained, and 543 ASVs were shared among the three groups. The P1 group had the richest ASVs while the N group had the least (Figure 1(a)). After annotation, a total of 30 phyla, 226 families, 485 genera, and 657 species were found. The sequence dilution curves of the three groups' samples indicated that the sequencing depths were reasonable (Figure 1(b)).

### 3.3. Abundance and Diversity of Vaginal Microbiome

Species abundance and diversity of the vaginal microbiome were assessed with Chao1 and Shannon indices, respectively. Chao1 index representing the average species abundance in the *P*1 group (105.01 ± 45.38) was significantly higher than that in the *N* group (80.18 ± 35.74, *P* < 0.01) and *P*2 group (90.33 ± 63.95, *p* < 0.05) (Figure 1(c)). Shannon index that comprehensively assessed the abundance and evenness of species in the *P*1 group (1.61 ± 1.07) was significantly higher than that in the *N* group (1.15 ± 0.74, *P* < 0.05), but there was no significant difference between the *P*1 group and *P*2 group (1.31 ± 1.06, *P* > 0.05) (Figure 1(d)).

### 3.4. Structure and *β*-diversity of the Vaginal Microbiome

**Figure 2:**
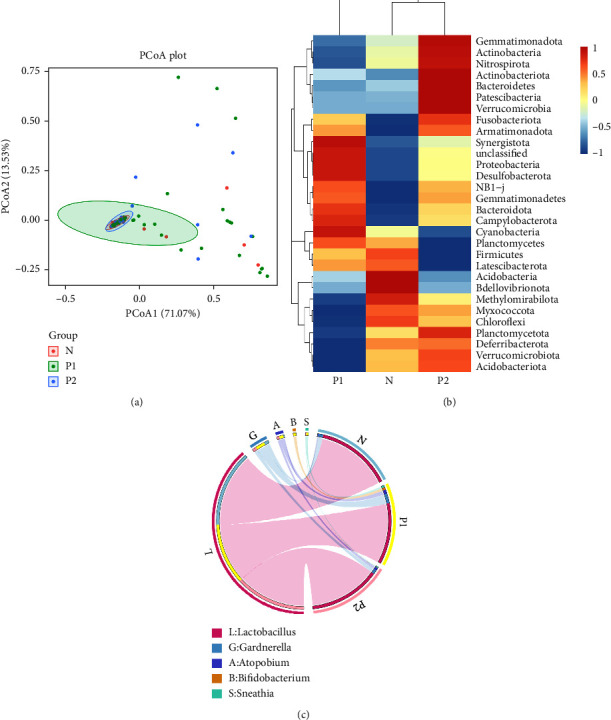
Structure and *β*-diversity of the vaginal microbiome. (a) *β*-diversity among groups. PCoA was performed based on the weight-UniFrac distance. The percentages of the abscissa and ordinate represent the interpretation of the sample gap by this dimension. (b) Heat map of relative abundance of TOP30 richest phyla among different groups. (c) Circle diagram of the relative abundance of TOP5 richest genera among different groups.

PCoA (principal co-ordinates analysis) was performed using a weighted UniFrac matrix (Figure 2(a)), and the two principal components explained 71.07% and 13.53%, respectively. The *N* group overlapped with the *P*2 group, and both *N* group and *P*2 group overlapped with the *P*1 group. At the phylum level, the top five phyla in the three groups were *Firmicutes*, *Actinobacteriota*, *Fusobacteriota*, *Bacteroidota*, and *Proteobacteria* (Figure 2(b)). However, the relative abundance of each phylum varied among groups. The relative abundances of *Firmicutes* in *N*, *P*1, and *P*2 groups were 92.09%, 76.84%, and 88.52%, respectively (*P*=0.003). The relative abundances of *Actinobacteriota* in the three groups were 7.19%, 19.4%, and 8.52% (*P*=0.009). The relative abundance of *Fusobacteriota* in the *P*1 group (2.34%) was higher than that in the *N* group (0.20%) and in the *P*2 group (1.17%), but there was no statistical significance (*p*=0.365). The relative abundance of *Bacteroidota* in the *P*2 group (1.03%) was higher than that in the *N* group (0.11%) and in the *P*1 group (0.52%), but there was no statistical significance (*P*=0.087). There was no significant difference in the relative abundance of *Proteobacteria* among three groups.

At the genus level, the top five genera in the three groups are shown in Figure 2(c). The genera with relative abundance ≥1% in the three groups are shown in Table 2. *Lactobacillus* was the dominant genus in all three groups, with significant differences among groups. It is worth noting that *Shuttleworthia* was only present in the *P*1 (0.01%) and *P*2 groups (1.01%) (*P*1 group vs. *N* group, *P*=0.014; *P*2 group vs. *N* group, *P*=0.004; *P*1 group vs. *P*2 group, *P*=0.383).

**Figure 3:**
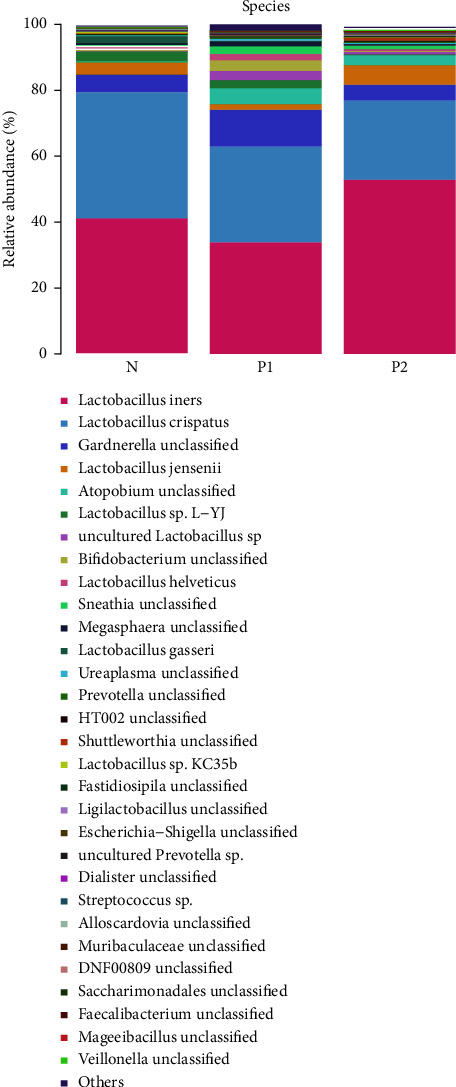
Column stacking diagram of relative abundance of top 30 species of the vaginal microbiome among the three groups.

At the species level of *Lactobacillus* (Figure 3 and Table 2), *Lactobacillus iners* were the most abundant bacteria among the three groups and was also the dominant bacteria in the *P*2 group (*P*2 vs. *P*1, *P*=0.024). The relative abundance of *Lactobacillus crispatus* in the three groups showed a decreasing trend (*P*2 group vs. *N* group, *P*=0.020). The relative abundance of *Lactobacillus gasseri* and *Lactobacillus jensenii* was significantly lower than *Lactobacillus iners and Lactobacillus crispatus*. However, there was no significant difference in *Lactobacillus gasseri* and *Lactobacillus jensenii* among groups.

### 3.5. Identification of Signature Vaginal Microbes

**Figure 4:**
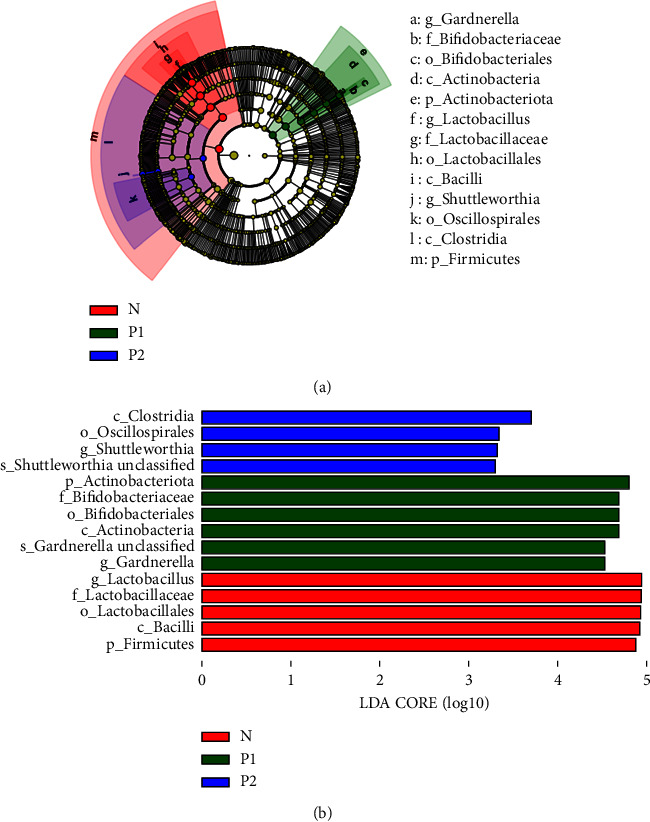
Identification of signature vaginal microbes. (a) Phylogenetic tree of species. Different circle layers radiate from the inside to the outside to represent the seven taxonomic levels of kingdom, phylum, class, order, family, genus, and species. Each node represents a species classification at this taxonomic level. The higher the abundance of the species, the larger the node. The yellow node means that the species has no significant difference among groups, and other color node means that the species has a significant difference among groups. (b) Histogram shows the LEfSe (LDA effect size) analysis of significantly differential bacteria from phylum to species level among groups (LDA score ≥ 3.0).

Due to the limitations of 16S rDNA amplicon sequencing technology, not all bacteria can be typed to the species level. Thus, we performed the analysis from the phylum to the genus level. Phylogenetic tree of species (Figure 4(a)) showed the different microbes among the three groups from phylum to genus level. Analysis by LEfSe showed that there were signature microbes in each group (*P* < 0.05, LDA score ≥ 3.0) (Figure 4(b)). The key genus with the most significant difference in the *N* group was *Lactobacillus*, while in the *P*1 group was *Gardnerella*, and in the *P*2 group was *Shuttleworthia.*

### 3.6. Function Analysis of Vaginal Microbes

**Figure 5:**
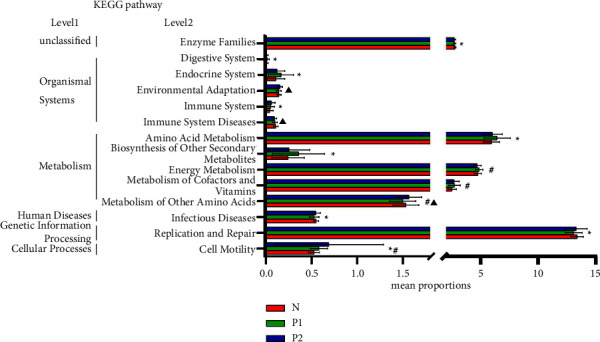
Differences in vaginal microbial function based on the KEGG pathway. The *t*-test was used for the pairwise comparison between groups. ^∗^Comparison between the *N* group and the *P*1 group, *P* < 0.05; ^#^Comparison between the *N* group and the *P*2 group, *P* < 0.05; ^▲^Comparison between the *P*1 and the *P*2 group, *P* < 0.05.

PICRUSt2 (Phylogenetic Investigation of Communities by Reconstruction of Unobserved States) based on the KEGG (Kyoto Encyclopedia of Genes and Genomes) database was used to predict the function of microbes. When comparing between the groups, we found out the metabolic and functional differences caused by vaginal microbes. At the KEGG pathway level 2, there were 39 functional gene pathways with different microbial gene abundances in the three groups, and 14 pathways were significantly different among groups (Figure 5), mainly on metabolism and gene information processing. Compared with the *P*1 group, the proportion of functional gene pathways of enzyme family, infectious diseases, replication, and repair in the *N* group was significantly increased. The amino acid metabolism, energy metabolism, metabolism of cofactor, and vitamin and cell motility functional gene pathways in the *P*1 group were significantly increased compared with the *N* group. By contrast, the genes of immune system diseases and metabolic pathways in the *P*2 group were also significantly richer than those in the *P*1 group.

## 4. Discussion

This is the first study to use 16S rDNA amplicon sequencing technology based on the NovaSeq 6000 platform to explore the characteristics of vaginal microbes in healthy and HPV-infected females of reproductive-age in Xinjiang, China. The sequencing results were processed by DADA2. The advanced sequencing platforms and processing methods make the obtained data more accurate [14]. Among asymptomatic females of reproductive-age, vaginal microbes are dominated by *Lactobacillus* spp. Ravel et al. said that *Lactobacillus iners* were more common in Asian females [16]. Consistently, this study found that *Lactobacillus iners* had the highest proportion in vaginal microbes of healthy females in Xinjiang, China. However, the proportion was no more than 50%.

We found that compared with healthy females, the species abundance and diversity of vaginal microbes in HPV-infected patients without LSIL were significantly increased, which suggests that there are alterations in vaginal microbes in the early stage of HPV infection. This is consistent with the findings of majority studies [17–19] but not consistent with that by Wu et al. [20]. This may be caused by the regional differences of the participants. Moreover, we found that in the early stage of HPV infection, vaginal microbes were mainly characterized by a decreasing proportion of *Lactobacillus crispatus* and an increasing proportion of anaerobic bacteria, especially the *Gardnerella,* which is similar to the results of Yang et al. [21]. The structure of vaginal microbiome in HPV-positive females was similar to bacterial vaginosis. The latter has been reported to be associated with sexually transmitted diseases (such as HIV), preterm labor, pelvic inflammatory disease, etc. [22]. Such vaginal microbiome structure may enhance the susceptibility of cervical epithelium to HPV.

Our study showed that the proportion of HPV multiple infections in the *P*2 group was significantly increased. This indicates that multiple infections may promote lesion development, as we all know. However, the vaginal microbial abundance and diversity of the *P*2 group were slightly higher than those in the *N* group, without significant difference. This is similar to the results of a previous study [23]. Nevertheless, Chen et al. showed that the difference between LSIL group and healthy group was obvious [6]. They included postmenopausal females in their study. Thus, the difference may be related to the different subjects. Additionally, there were significant differences in microbial composition in the *P*2 group compared to that in the *N* group, with the highest abundance of *Lactobacillus iners* and the lowest abundance of *Lactobacillus crispatus* in the three groups, which is similar to the findings of Chao et al. in persistently infected HPV patients [24]. This is also consistent to the meta-analysis by Norenhag et al. [5], which concluded that compared with vaginal microbiota dominated by *Lactobacillus crispatus*, precancerous lesions are more likely to occur in patients with *Lactobacillus iners*-dominated vaginal microbiota.

This study also found that the unique microbe of the vaginal microbiota in the P2 group was the genus *Shuttleworthia*. *Shuttleworthia* is a Gram-positive anaerobic bacilli. Downes first reported the isolation of *Shuttleworthia* and *Shuttleworthia satelles* from human oral secretions in 2002 [25]. Then, investigators have noticed that they were enriched in the oral microbes of periodontitis [26] and childhood caries [27]. Recently, Eun et al. showed that in patients with oral squamous cell carcinoma, the level of *Shuttleworthia* in the oral microbiota of patients with lymph node metastasis was significantly enriched [28], indicating that it may be involved in the malignant transfer. Recently, this genus has also been found in the microbes of the female reproductive tract. In 2019, Onywera et al. reported that *Shuttleworthia* was common in bacterial vaginosis [29]. In 2021, Yuan and colleagues also showed that eight bacterial genera, including *Shuttleworthia*, were strongly associated with bacterial vaginosis [30]. The association of *Shuttleworthia* with HPV infection and cervical lesions is also being studied. Chorna et al. performed metagenomic analysis of vaginal samples from 19 Puerto Rican females, including 8 HPV-negative samples, and 11 high-riskHPV-positive samples. Significant differences were presented in microbial diversity, with *Atopobium* being enriched in the high-riskHPV-positive group and *Lactobacillus* increasing in the HPV-negative group with a low abundance of *Lactobacillus iners* and *Shuttleworthia* [31]. Chen et al. conducted a study on the characterization of vaginal microbiota in 229 samples from different grades of cervical lesions in Shanghai, China, and they found that, at the family level, *Prevotella timonensis*, *Shuttleworthia*, and *Streptococcus* were associated with HSIL [6]. However, Liu et al. performed 16S rRNA amplicon analysis of vaginal secretions from 122 female patients in Beijing, China and concluded that *Shuttleworthia* was a beneficial bacterium for high-risk HPV infection [32]. The discrepancy may be related to the different inclusion criteria of participants, regions, and sample sizes. The participants of this study were females of reproductive age who lived in Xinjiang, China. It showed that the genus *Shuttleworthia* was significantly enriched in females who were infected with HPV and were with LSIL. The increase of *Shuttleworthia* may accelerate cervical epithelial lesions, but the specific pathogenic mechanism remains to be elucidated.

In this study, the PICRUSt functional prediction showed that the abundance of pathways of energy metabolism, metabolism of amino acids, biosynthesis of secondary metabolites, and metabolism of cofactors and vitamin of vaginal microbiome in the *P*1 group was significantly higher than those in the *N* group. However, the abundance of pathway of replication and repair in *P*1 and *P*2 groups was significantly lower than that in the *N* group. These results are consistent with those by Yang et al. [21], indicating that inordinate vaginal microbiome may facilitate HPV replication and transmission and contribute to HPV integration into host genes. Interestingly, there was a significant increase in cell motility function gene pathways in *P*1 and *P*2 groups, which may be associated with good outcomes after HPV infection according to Usyk et al.'s study [19].

There are some limitations in this study. First, this is a cross-sectional study, which cannot clarify the causal relationship between different bacterial genera and clinical diagnosis. Second, clinical indicators that might be related to HPV infection, such as inflammation index, vaginal PH, and serum estrogen level, were not included for further analysis. Third, the sample size was small. Further studies are warranted.

## 5. Conclusions

In conclusion, this study revealed that the vaginal microbiota of asymptomatic females of reproductive-age in Xinjiang was dominated by *Lactobacillus*, among which the relative abundance of *Lactobacillus crispatus* was higher in healthy females. The significant increase in *Gardnerella* may enhance the susceptibility of cervical epithelium to HPV infection. The predominance of *Lactobacillus iners* and the presence of *Shuttleworthia* may be a signature for HPV-infected females with LSIL.

## Figures and Tables

**Table 1:** Demographic characteristics of participants.

	Total	*N* group	*P*1 group	*P*2 group	*P* value
No. of cases (*n*)	135	43	58	34	
Age (years) (mean ± SD)/range	32.27 ± 5.88/(22–47)	33.65 ± 5.25/(25–45)	31.36 ± 5.86/(22–46)	32.06 ± 6.48/(23–47)	0.833^a^

Ethnicity, *n* (%)					
Han	119 (88.1)	35 (81.4)	53 (91.4)	31 (91.2)	0.318^b^
Others	16 (11.9)	8 (18.6)	5 (8.6)	3 (8.8)	

Body mass index (kg/m^2^), *n* (%)					
<18.5	16 (11.9)	5 (11.6)	7 (12.1)	4 (11.8)	0.993^b^
18.5–24	88 (65.2)	28 (65.1)	39 (67.2)	21 (61.8)	
24–28	27 (20.0)	9 (20.9)	10 (17.2)	8 (23.5)	
≥28	4 (3.0)	1 (2.0)	2 (3.4)	1 (2.9)	
No. of pregnancies (median [1/4–3/4 interquartile range])	2.0 (0–3)	2.0 (0–2)	1.0 (0–3)	1.5 (0.75–3.0)	0.556^d^
No. of births (median [1/4–3/4 interquartile range])	0.0 (0–1)	1.0 (0–1)	0 (0–1.25)	0.5 (0–2.0)	0.656^d^

Phase of menstrual cycle, *n* (%)					
Follicular phase	76 (56.3)	20 (46.5)	35 (60.3)	21 (61.8)	0.290^c^
Luteal phase	59 (43.7)	23 (53.5)	23 (39.7)	13 (38.2)	

Contraception, *n* (%)					
No contraception	26 (19.3)	8 (18.6)	11 (19.0)	7 (20.6)	0.900^b^
Condom	86 (63.7)	27 (62.8)	37 (63.8)	22 (64.7)	
IUD	12 (8.9)	4 (9.3)	5 (8.6)	3 (8.8)	
Other	11 (8.1)	4 (9.3)	5 (8.6)	2 (5.9)	

Age at first sex (years), *n* (%)					
≥20	113 (83.7)	39 (90.7)	47 (81.0)	27 (79.4)	0.304^b^
<20	22 (16.3)	4 (9.3)	11 (19.0)	7 (20.6)	

No. of sexual partners in lifetime, *n* (%)					
1–2	104 (77.0)	37 (86.0)	42 (72.4)	25 (73.5)	0.186^b^
3–5	30 (22.2)	6 (14.0)	16 (27.6)	8 (23.5)	
≥6	1 (0.7)	0 (0)	0 (0)	1 (2.9)	

Frequency of sex in the past year, *n* (%)					
≤1 time/week	104 (77.0)	34 (79.1)	43 (74.1)	27 (79.4)	0.956^b^
2–3 times/week	30 (22.2)	9 (20.9)	14 (24.1)	7 (20.6)	
≥4 times/week	1 (0.7)	0 (0)	1 (1.7)	0 (0)	

Smoking, *n* (%)					
No	100 (74.1)	34 (79.1)	46 (79.3)	20 (58.8)	0.111^b^
Active smoking	5 (3.7)	1 (2.3)	3 (5.2)	1 (2.9)	
Passive smoking	30 (22.2)	8 (18.6)	9 (15.5)	13 (38.2)	

HPV status, *n* (%)					
Positive for HPV16/18	17 (12.6)	0 (0)	15 (25.9)	2 (5.9)	0.014^be^
Positive for 12 other HPV types	54 (40.0)	0 (0)	34 (58.6)	20 (58.8)	
Positive for both HPV16/18 and 12 other HPV types	21 (15.6)	0 (0)	9 (15.5)	12 (35.3)	
Negative	43 (31.9)	43 (100)	0 (0)	0 (0)	

*Note.* (a) The ANOVA test with mean ± SD. SD, standard deviation. (b) Fisher's exact test with *n* (%) when there is a *n* < 5; otherwise, the Chi-square test. (c) *P* < 0.05 (in bold) represents statistical significance. (d) The Kruskal–Wallis test with median (1/4–3/4 interquartile range). (e) Comparing between *P*1 and *P*2. IUD, intrauterine device; HPV, human papillomavirus.

**Table 2:** Comparison of genera and species with relative abundance >1% among three groups (%).

	N	*P*1	*P*2	*P* value
*Lactobacillus*	89.87	72.14	84.35	0.000^#∆^
*Lactobacillus crispatus*	38.30	29.14	24.08	0.053^∗^
*Lactobacillus iners*	40.73	33.89	52.88	0.084^∆^
*Lactobacillus gasseri*	2.11	2.11	0.03	0.470
*Lactobacillus jensenii*	3.34	1.68	5.88	0.174
*Gardnerella*	5.31	11.12	4.79	0.003^#^
*Atopobium*	1.19	4.84	3.18	0.809
*Bifidobacterium*	0.18	3.23	0.28	0.490
*Sneathia*	0.20	2.34	1.02	0.452
*Megasphaera*	0.51	1.50	0.53	0.102
*Shuttleworthia*	0.00	0.01	1.01	0.012^∗#^

*Note.* The Kruskal–Wallis test was used. Compared between *N* group and *P*2 group, ^∗^*P* < 0.05; Compared between *P*1 group and *P*2 group, ^∆^*P* < 0.05; Compared between *N* group and *P*1 group, ^#^*P* < 0.05.

## Data Availability

The datasets used and/or analyzed during the current study are available from the corresponding authors upon reasonable request.

## References

[1] Sung H., Ferlay J., Siegel R. L. (2021). Global cancer statistics 2020: GLOBOCAN estimates of incidence and mortality worldwide for 36 cancers in 185 countries. *CA: A Cancer Journal for Clinicians*.

[2] Plummer M., Schiffman M., Castle P., Maucort‐Boulch D., Wheeler C. (2007). A 2-year prospective study of human papillomavirus persistence among women with a cytological diagnosis of atypical squamous cells of undetermined significance or low-grade squamous intraepithelial lesion. *The Journal of Infectious Diseases*.

[3] Castanheira C. P., Sallas M., Nunes R., Lorenzi N., Termini L. (2021). Microbiome and cervical cancer. *Pathobiology*.

[4] Andrade Pessoa Morales J., Marconi C., El-Zein M. (2022). Vaginal microbiome components as correlates of cervical human papillomavirus infection. *The Journal of Infectious Diseases*.

[5] Norenhag J., Du J., Olovsson M., Verstraelen H., Engstrand L., Brusselaers N. (2020). The vaginal microbiota, human papillomavirus and cervical dysplasia: a systematic review and network meta-analysis. *BJOG: An International Journal of Obstetrics and Gynaecology*.

[6] Chen Y., Qiu X., Wang W. (2020). Human papillomavirus infection and cervical intraepithelial neoplasia progression are associated with increased vaginal microbiome diversity in a Chinese cohort. *BMC Infectious Diseases*.

[7] Mitra A., MacIntyre D. A., Lee Y. S. (2015). Cervical intraepithelial neoplasia disease progression is associated with increased vaginal microbiome diversity. *Scientific Reports*.

[8] Lebeau A., Bruyere D., Roncarati P. (2022). HPV infection alters vaginal microbiome through down-regulating host mucosal innate peptides used by Lactobacilli as amino acid sources. *Nature Communications*.

[9] Burd E. M. (2003). Human papillomavirus and cervical cancer. *Clinical Microbiology Reviews*.

[10] Balasubramaniam S. D., Balakrishnan V., Oon C. E., Kaur G. (2019). Key molecular events in cervical cancer development. *Medicina (Kaunas)*.

[11] Nuovo G. J., De Andrade C. V., Wells S. I., Brusadelli M., Nicol A. F. (2018). New biomarkers of human papillomavirus infection in acute cervical intraepithelial neoplasia. *Annals of Diagnostic Pathology*.

[12] Zheng Q., chen X., Han R. (2021). HPV58 E7 protein expression profile in cervical cancer and CIN with immunohistochemistry. *Journal of Cancer*.

[13] Bhattarakosol P., Plaignam K., Sereemaspun A. (2018). Immunogold-agglutination assay for direct detection of HPV-16 E6 and L1 proteins from clinical specimens. *Journal of Virological Methods*.

[14] Xue Z., Kable M. E., Marco M. L. (2018). Impact of DNA sequencing and analysis methods on 16S rRNA gene bacterial community analysis of dairy products. *MSphere*.

[15] Callahan B. J., McMurdie P. J., Rosen M. J., Han A. W., Johnson A. J. A., Holmes S. P. (2016). DADA2: high-resolution sample inference from Illumina amplicon data. *Nature Methods*.

[16] Ravel J., Gajer P., Abdo Z. (2011). Vaginal microbiome of reproductive-age women. *Proceedings of the National Academy of Sciences of the United States of America*.

[17] Zhang Z., Li T., Zhang D. (2021). Distinction between vaginal and cervical microbiota in high-risk human papilloma virus-infected women in China. *BMC Microbiology*.

[18] Cheng L., Norenhag J., Hu Y. O. O. (2020). Vaginal microbiota and human papillomavirus infection among young Swedish women. *NPJ Biofilms Microbiomes*.

[19] Usyk M., Zolnik C. P., Castle P. E. (2020). Cervicovaginal microbiome and natural history of HPV in a longitudinal study. *PLoS Pathogens*.

[20] Wu M., Gao J., Wu Y. (2020). Characterization of vaginal microbiota in Chinese women with cervical squamous intra-epithelial neoplasia. *International Journal of Gynecological Cancer*.

[21] Yang Q., Wang Y., Wei X. (2020). The alterations of vaginal microbiome in HPV16 infection as identified by shotgun metagenomic sequencing. *Frontiers in Cellular and Infection Microbiology*.

[22] Muzny C. A., Laniewski P., Schwebke J. R., Herbst-Kralovetz M. M. (2020). Host-vaginal microbiota interactions in the pathogenesis of bacterial vaginosis. *Current Opinion in Infectious Diseases*.

[23] Huang X., Li C., Li F., Zhao J., Wan X., Wang K. (2018). Cervicovaginal microbiota composition correlates with the acquisition of high-risk human papillomavirus types. *International Journal of Cancer*.

[24] Chao X., Sun T., Wang S. (2020). Research of the potential biomarkers in vaginal microbiome for persistent high-risk human papillomavirus infection. *Annals of Translational Medicine*.

[25] Downes J. (2002). Shuttleworthia satelles gen. nov., sp. nov., isolated from the human oral cavity. *International Journal of Systematic and Evolutionary Microbiology*.

[26] Colombo A. P. V., Boches S. K., Cotton S. L. (2009). Comparisons of subgingival microbial profiles of refractory periodontitis, severe periodontitis, and periodontal health using the human oral microbe identification microarray. *Journal of Periodontology*.

[27] Wang Y., Wang S., Wu C. (2019). Oral microbiome alterations associated with early childhood caries highlight the importance of carbohydrate metabolic activities. *mSystems*.

[28] Eun Y. G., Lee J. W., Kim S. W., Hyun D. W., Bae J. W., Lee Y. C. (2021). Oral microbiome associated with lymph node metastasis in oral squamous cell carcinoma. *Scientific Reports*.

[29] Onywera H., Williamson A. L., Mbulawa Z. Z., Coetzee D., Meiring T. L. (2019). Factors associated with the composition and diversity of the cervical microbiota of reproductive-age Black South African women: a retrospective cross-sectional study. *PeerJ*.

[30] Yuan D., Chen W., Qin J., Shen D., Qiao Y., Kong B. (2021). Associations between bacterial vaginosis, candida vaginitis, trichomonas vaginalis, and vaginal pathogenic community in Chinese women. *American Journal of Translational Research*.

[31] Chorna N., Romaguera J., Godoy-Vitorino F. (2020). Cervicovaginal microbiome and urine metabolome paired analysis reveals niche partitioning of the microbiota in patients with human papilloma virus infections. *Metabolites*.

[32] Liu J., Luo M., Zhang Y., Cao G., Wang S. (2020). Association of high-risk human papillomavirus infection duration and cervical lesions with vaginal microbiota composition. *Annals of Translational Medicine*.

